# Supplementary material for: The Composites of PCL and Tetranuclear Titanium(IV)-oxo Complexes as Materials Exhibiting the Photocatalytic and the Antimicrobial Activity
Source: Int J Mol Sci. 2021 Jun 29;22(13):7021. doi: 10.3390/ijms22137021 (PMC8268633; doi:10.3390/ijms22137021)
Supplement: Supplementary file 1 [file ijms-22-07021-s001.zip › Figure S1.pptx]

## Slide 1
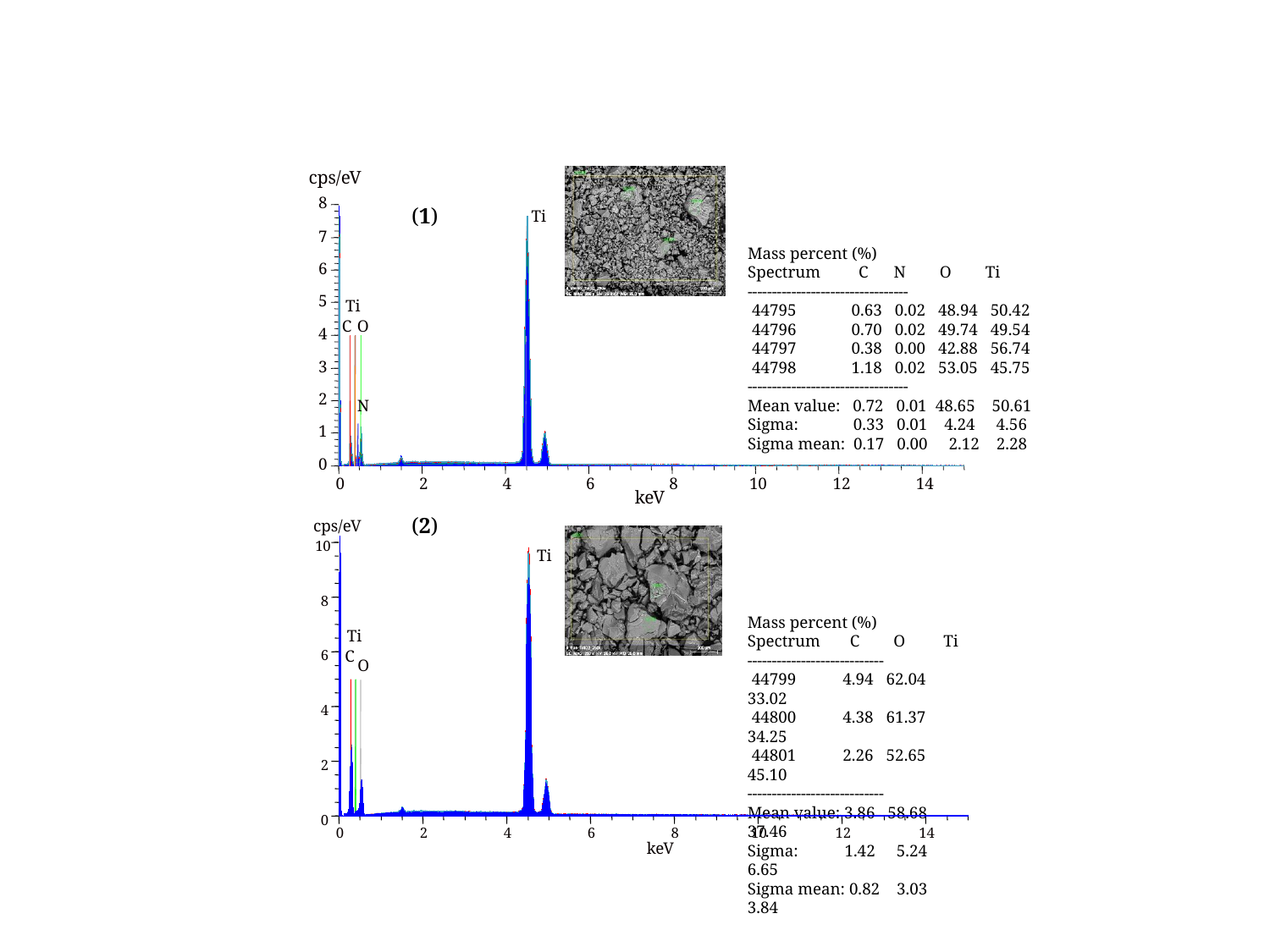

cps/eV
8
 Ti
7
6
5
 Ti
 C
 O
4
3
2
 N
1
0
0
2
4
6
8
10
12
14
keV
(1)
Mass percent (%)
Spectrum C N O Ti
---------------------------------
 44795 0.63 0.02 48.94 50.42
 44796 0.70 0.02 49.74 49.54
 44797 0.38 0.00 42.88 56.74
 44798 1.18 0.02 53.05 45.75
---------------------------------
Mean value: 0.72 0.01 48.65 50.61
Sigma: 0.33 0.01 4.24 4.56
Sigma mean: 0.17 0.00 2.12 2.28
(2)
 cps/eV
10
 Ti
8
Mass percent (%)
Spectrum C O Ti
----------------------------
 44799 4.94 62.04 33.02
 44800 4.38 61.37 34.25
 44801 2.26 52.65 45.10
----------------------------
Mean value: 3.86 58.68 37.46
Sigma: 1.42 5.24 6.65
Sigma mean: 0.82 3.03 3.84
 Ti
 C
6
 O
4
2
0
0
2
4
6
8
10
12
14
keV
