# Supplementary figures and images for: The Composites of PCL and Tetranuclear Titanium(IV)-oxo Complexes as Materials Exhibiting the Photocatalytic and the Antimicrobial Activity
Source: Int J Mol Sci. 2021 Jun 29;22(13):7021. doi: 10.3390/ijms22137021 (PMC8268633; doi:10.3390/ijms22137021)

## Slide 1
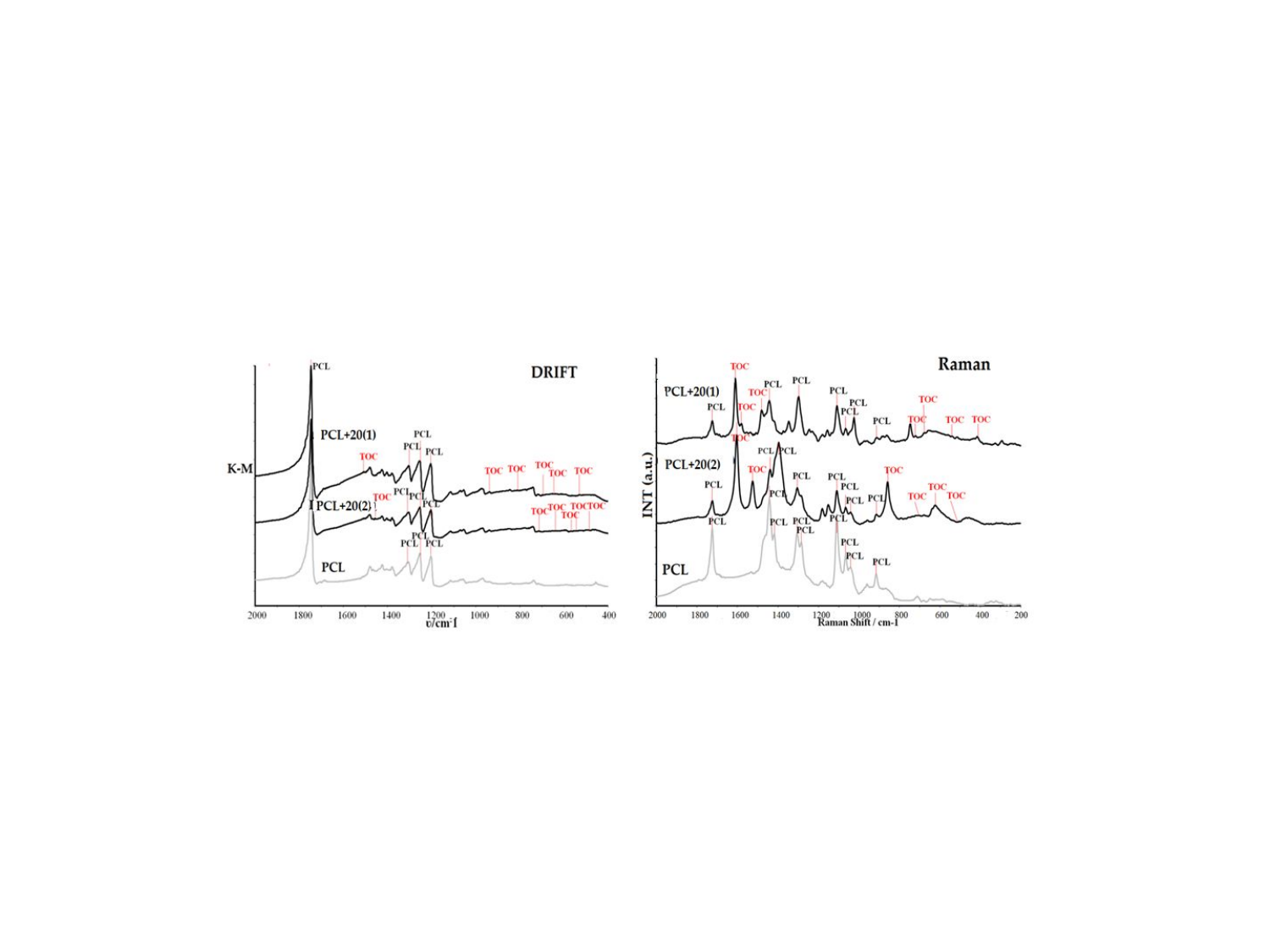

Supplement: Supplementary file 1 [file ijms-22-07021-s001.zip › Figure S2.pptx]
